# Translation, validation and psychometric properties of the Dutch version of the Inflammatory Bowel Disease-Fatigue (IBD-F) self-assessment scale

**DOI:** 10.1186/s41687-023-00642-3

**Published:** 2023-10-30

**Authors:** Annemay M. H. Stoker, Angélique Gruters, Mirjam C. M. van der Ende-van Loon, Debby Postulart, Wladyslawa Czuber-Dochan, Lennard P. L. Gilissen

**Affiliations:** 1 Department of Gastroenterology and Hepatology, Catharina Hospital Eindhoven, Michelangelolaan 2, Eindhoven, 5632 EJ Netherlands; 2 Department of Medical Psychology, Catharina Hospital Eindhoven, Eindhoven, Netherlands; 3 Fontys University of Applied Sciences, Tilburg, Netherlands; 4 Florence Nightingale Faculty of Nursing, Midwifery & Palliative Care, King’s College London, London, UK

**Keywords:** Inflammatory bowel diseases, Fatigue, Patient reported outcome measures, Psychometrics, Translation, Dutch

## Abstract

**Background:**

In patients with inflammatory bowel disease (IBD), a symptom with major impact on health-related quality of life is fatigue. To assess fatigue and conduct research regarding fatigue in IBD patients, a validated disease specific assessment tool is required. The aim of this study was to translate the Inflammatory Bowel Disease Fatigue patient self-assessment scale (IBD-F) into Dutch and to validate this translated scale in a Dutch IBD population.

**Methods:**

The study comprised three phases. In phase 1, the original IBD-F was translated into Dutch. Phase 2 comprised a pilot-test of the pre-final Dutch IBD-F to assess content validity by applying a semi-structured interview design. In phase 3, construct validity, internal consistency and test-retest reliability were assessed using a cross-sectional design.

**Results:**

Phase 1 resulted in the pre-final version of the Dutch IBD-F. After five semi-structured interviews with IBD patients in phase 2, minor adjustments were made which resulted in the final version of the Dutch IBD-F. Evaluation of this final version in 133 IBD patients showed adequate psychometric properties: good convergent validity with the Multidimensional Fatigue Inventory subscales (Spearman’s *r* 0.57–0.86) and excellent internal consistency (Cronbach’s alpha 0.94 for Section I and 0.97 for Section II). Test-retest reliability in 102 patients was shown to be good (Section I ICC 0.85 (95% CI 0.79–0.90) and Section II ICC 0.88 (95% CI 0.83–0.92)).

**Conclusions:**

The thorough translation process resulted in a comprehensible, valid and reliable version of the Dutch IBD-F. Convergent validity with the MFI-20 appeared to be good. This study found excellent internal consistency and good test-retest reliability.

**Supplementary Information:**

The online version contains supplementary material available at 10.1186/s41687-023-00642-3.

## Background

In patients with inflammatory bowel disease (IBD), one of the most impacting symptoms that needs to be managed to improve health-related quality of life (HRQoL) is fatigue. IBD refers to conditions which are characterized by chronic inflammation of the gastrointestinal tract including Crohn’s disease (CD), ulcerative colitis (UC) and IBD-unspecified (IBD-U). Fatigue is highly prevalent in patients with active disease (72%). However, it is also frequently reported by patients in remission (47%) [1]. It has a substantial negative impact on HRQoL in patients with IBD and it is perceived by patients as distressing and impairing in daily life functioning [2–5]. Concomitantly, due to the complexity and multifactorial aspect of fatigue, this is one of the most challenging symptoms to manage in clinical IBD practice [6].

Fatigue is often defined as ‘a sense of continuing tiredness, with periods of sudden and overwhelming lack of energy or a feeling of exhaustion that is not (fully) relieved after rest or sleep’ [5]. This clinical symptom is, together with pain, diarrhoea and rectal bleeding, one of the most frequently reported symptoms by patients with IBD [2, 7, 8]. Healthcare providers reported the requirement for a measurement tool to evaluate fatigue in clinical practice in order to improve HRQoL of patients with IBD and to reduce the burden of this debilitating symptom [9]. Furthermore, the Nurses European Crohn’s and Colitis Organisation (N-ECCO) identified IBD fatigue as one of the top research priorities [10]. Though, a validated IBD specific assessment tool to assess the level and impact of fatigue is crucial since available general patient-reported outcome measures (PROMs) to evaluate HRQoL (e.g. the inflammatory bowel disease questionnaire (IBDQ)) may obscure fatigue symptoms when overall scores improve [6, 11].

To measure fatigue, multiple self-assessment tools are available and applied in IBD research: Functional Assessment of Chronic Illness Therapy-Fatigue (FACIT-F), Fatigue Questionnaire (FQ), Multidimensional Assessment Fatigue (MAF), Multidimensional Fatigue Inventory (MFI-20) and the Inflammatory Bowel Disease-Fatigue patient self-assessment scale (IBD-F) [5, 12–15]. The FQ, MAF, MFI-20 and FACIT-F are assessment tools with adequate measurement properties. However, they are not validated in patients with IBD or not developed particularly for IBD nor validated in a Dutch IBD population [16, 17]. The IBD-F is the only assessment tool specifically developed to measure fatigue in patients with IBD. These patients with IBD preferred the IBD-F since it best reflects their experience of fatigue [5]. Furthermore, a recent systematic review on PROMs concerning fatigue in patients with IBD, recommended the use of the IBD-F to evaluate fatigue in this population [18]. This self-assessment scale was developed in the United Kingdom and was translated into different languages and validated in multiple countries [19–23]. The scale comprises three sections: 5 items in Section I evaluating the level of fatigue, 30 items in Section II assessing the global impact of fatigue in the past two weeks and 5 open-ended questions in Section III which can be used as an aid to discuss IBD fatigue during clinical consultations. The internal consistency of the original IBD-F is excellent (Cronbach’s alpha 0.91 for Section I and Cronbach’s alpha 0.98 for Section II) and the test-retest reliability is proven to be adequate (ICC 0.74 for Section I and ICC 0.83 for Section II) [5].

To date the IBD-F self-assessment scale is not available in Dutch. The lack of an IBD-specific Dutch self-assessment tool, complicates the evaluation of fatigue in clinical practice. To this day, general HRQoL questionnaires are administered annually. However, in clinical practice, patients remark that the burden of fatigue is not fully addressed in these questionnaires. Furthermore, to adequately conduct research regarding this debilitating symptom in a Dutch population, a well validated instrument is essential. To use a measurement tool in a language that is different to the language that the original tool was developed in, a thorough translation process is needed. Besides, it cannot be assumed that this translated version holds the same measurement properties as the original tool does. Therefore, to assess the psychometric properties after translation, a validation study must be performed [24–26].

The aim of this study was to translate IBD-F patient self-assessment scale to the Dutch language and to validate this translated scale by assessing the psychometric properties, including content and construct validity, internal consistency and test-retest reliability in adult patients with IBD in the Netherlands.

## Methods

This cross-cultural validation study was conducted in a teaching hospital in the south of the Netherlands (Catharina Hospital, Eindhoven) and comprised three phases. Guidelines from Beaton et al. (2000) for the process of cross-cultural adaptation of self-report measures, standards from De Vet et al. (2020) and the COSMIN guidelines were applied [24–27]. Prior to the start of the study, permission was obtained from the author of the original IBD-F. The study was approved by the local ethics committee (MEC-U) (nWMO-2021.111). All participating patients provided written informed consent prior to enrolment.

### Phase 1

**Fig. 1:**
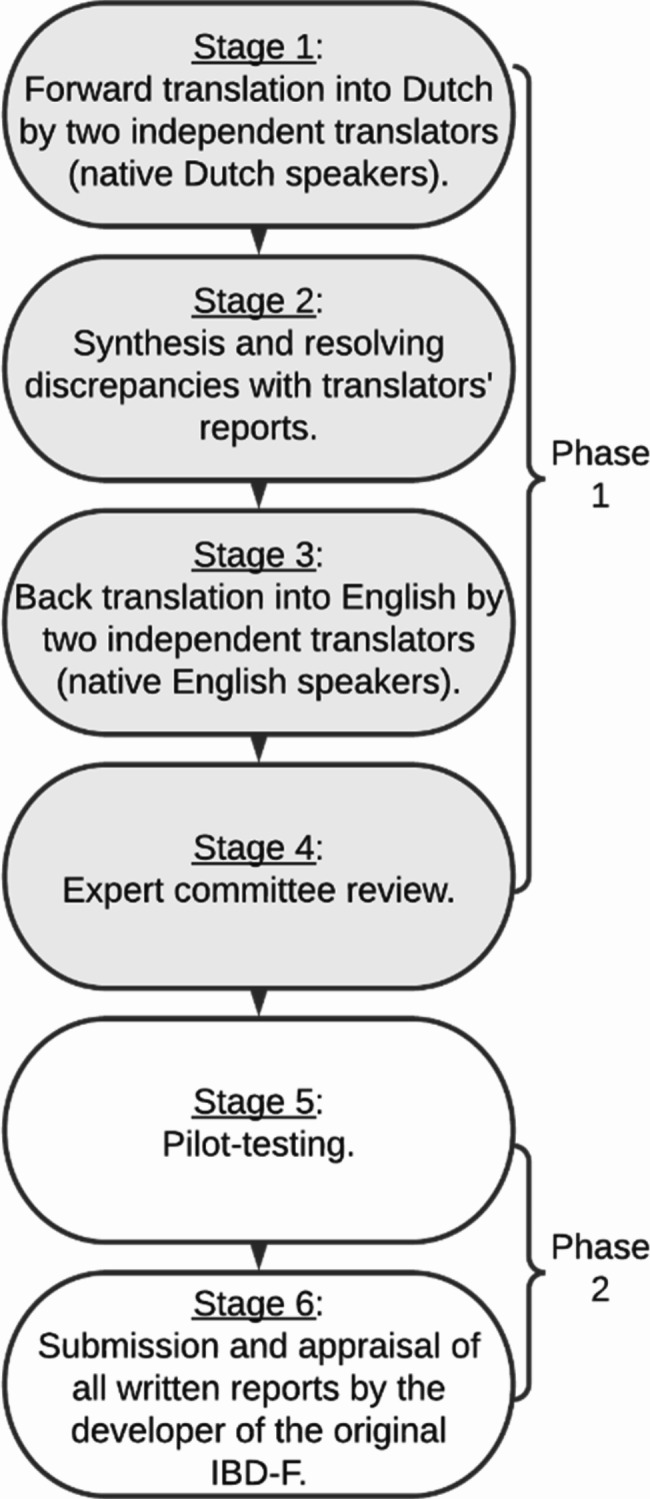
Translation process according guidelines from beaton et al. (2000) [25]

Phase 1 comprised the translation of the original IBD-F into Dutch. The first four out of six stages of cross-cultural adaptation recommended by Beaton et al. (2000) were performed, see Fig. 1 [25].

Both forward translators (A and B) were native Dutch speakers. Translator A is a medical psychologist with expertise in fatigue, whereas translator B is a language expert. The results of both forward translations were combined in one synthesized version. Discrepancies were discussed, resolved and carefully documented by both translators and the principal researcher (AS).

Subsequently, the synthesized version was translated back into English by two independent native English speakers (C and D). Translator C is a language expert. Translator D is health scientist with no expertise on fatigue. Translators C and D were blinded for the original scale. Finally, an expert committee consisting of translators and researchers (a language expert with a Bachelor of Arts in English Language and Culture and Master of Science in developmental linguistics, a gastroenterologist and two nurse practitioners with expertise on IBD, a medical psychologist with expertise on fatigue and an epidemiologist) reviewed all written reports, reached consensus on discrepancies and agreed on the pre-final version of the Dutch IBD-F.

### Phase 2

Phase 2 comprised pilot-testing of the Dutch IBD-F version by applying stage five and six of the guidelines from Beaton et al. (2000), see Fig. 1 [25]. To assess the comprehensibility of a scale, a sample size of 4–6 patients is considered to be adequate when using a qualitative method and achieving data saturation is considered to be more important than the magnitude of the sample size [26]. Five interviews were scheduled. If after five interviews data saturation was not achieved according to the expert committee, five more interviews would be conducted. Patients were purposively selected considering age, gender, disease type, and educational level to aim for heterogeneity in the sample. Patients were eligible for inclusion if they were 18 years or older and had both clinical and endoscopic diagnosis of IBD for at least two weeks prior to the study participation. Patients were excluded if they were unable to understand the study procedures or self-assessment instrument due to illiteracy or inability to speak and understand Dutch.

Patients were requested to complete the pre-final version of the Dutch IBD-F in the presence of the principal researcher (AS). Comprehensibility, relevance and completeness of the scale was assessed using the Three Step Test Interview (TSTI) method [24, 28]. See appendix 1 for the interview guide. The audio recordings of the interviews were transcribed, and all comments and suggestions were summarized. The findings were presented to the expert committee for final review, discussion and approval. To complete the sixth stage of the guidelines from Beaton et al. (2000), the developer of the original IBD-F performed a process audit by reviewing all written reports [25].

### Phase 3

#### Study design

Phase 3 comprised the assessment of the psychometric properties (validity and reliability) of the Dutch IBD-F using a cross-sectional survey and test-retest design.

#### Participants

Regarding validation studies, a minimum sample size of 50 participants is recommended, however larger samples of over 100 participants are preferred [24, 27]. Based on a clinical expert’s opinion, a response rate of 30% was estimated. Hence, a random sample of 300 out of a total of 1359 outpatient IBD-patients was selected. These 300 patients were assessed on the same inclusion and exclusion criteria as applied in phase 2. Patients who previously participated in phase 2 were excluded for the participation in phase 3. Patients eligible for participation received a postal study invitation including a patient information form and an informed consent form. A reminder letter was sent after three weeks.

#### Measurement tools

Fatigue was assessed by Section I and II of the Dutch IBD-F scale and by the MFI-20. The answer options of Section I of the IBD-F range from 0 (no fatigue or none of the time) to 4 (severe fatigue or all of the time) and the answer options of Section II are: 0 = none of the time, 1 = a little bit of the time, 2 = some of the time, 3 = most of the time and 4 = all of the time. Section III was not administered in phase 3 of the current study because of the free text answer options. The scoring system of the original IBD-F scale was applied. Sum scores for Section I and Section II were calculated separately. Possible sum scores in Section I ranged from 0 to 20 with a score of 0 indicating no fatigue. Sum scores of Section II were calculated by the following formula: *adjusted score = actual score / (120 – number of ‘N/A’ x 4) x 120* [5]. Possible sum scores in Section II range from 0 to 120 with higher scores indicating higher levels of fatigue [5, 29].

The MFI-20 was completed with the aim of assessing convergent validity with the IBD-F as a part of the construct validity and to compare the psychometric properties with the psychometric properties of the Dutch IBD-F. The MFI-20 was considered to be the most suitable scale to measure convergent validity since it is one of the most frequently used fatigue-scales in IBD research and it is available in Dutch [6, 15]. The MFI-20 is a self-assessment tool consisting of 20 items to measure fatigue in five subscales. Sum scores are calculated for each subscale individually and range from 0 to 20 per subscale. Higher scores indicate a higher level of fatigue. The MFI-20 appears to have good internal consistency with a Cronbach’s alpha coefficient of 0.84 [15].

To assess current disease activity, the Harvey-Bradshaw Index (HBI) for patients with Crohn’s disease (CD) and the Simple Clinical Colitis Activity Index (SCCAI) for patients with ulcerative colitis (UC) and inflammatory bowel disease unclassified (IBD-U) were administered. For both HBI and SCCAI, a score of ≤ 4 was classified as quiescent disease and a score of ≥ 5 is considered to indicate active disease. The HBI and SCCAI were selected to assess current disease activity because they are frequently used in IBD research, provide a clear identification of current disease activity, are easy to self-administer by patients and no invasive tests are needed [30, 31].

Patients completed the three scales (IBD-F, MFI-20 and HBI/SCCAI) twice: on baseline and three weeks later. They completed the scales at home and they were given the choice to complete the scales either electronically or on paper. Clinical and demographic characteristics were deducted from patients’ files. All data were filed in a digital case report form using Cloud9 Software® ResearchManager®, Deventer, the Netherlands.

#### Assessments and analysis

All data were analysed using IBM® SPSS® Statistics for Windows version 28.0., Armonk, New York. Distribution of data was assessed using the Kolmogorov-Smirnov test. Non-normally distributed continuous data was summarized by the median and range whereas normally distributed data was expressed as mean and standard deviation (SD). Categorical variables were expressed as frequencies and percentages. Consistent with the validation study of the original English IBD-F scale, individual items of the Dutch IBD-F scale as well as total sum scores of the IBD-F and MFI-20 were regarded as continuous variables. Individual items of the MFI-20 were considered to be categorical values [5, 15].

Construct validity, internal consistency and test-retest reliability were assessed. Construct validity was assessed by evaluation of the convergent validity of the Dutch IBD-F and the MFI-20 using Spearman’s *r*. Based on earlier research, a moderate correlation (Spearman’s *r* from 0.45 to 0.65) between the IBD-F and MFI-20 subscales was expected [5]. The highest correlation was assumed between Section I of the IBD-F and the subscale ‘general fatigue’ of the MFI-20 because these two parts both aim to assess the level of overall fatigue. Compared to the assumed correlation between the IBD-F Section I and the MFI-20 ‘general fatigue’ subscale, a lower correlation was expected between Section II of the IBD-F and the other subscales of the MFI-20 because those parts measure different aspects of fatigue. Convergent validity was considered to be adequate if at least 75% of the stated hypotheses prove to be correct or if a minimum correlation coefficient of 0.50 with the MFI-20 subscales was found [32, 33].

Reliability of the Dutch IBD-F was tested by evaluating internal consistency and through a test and retest three weeks after baseline. A period of three weeks was considered to be appropriate because this period was deemed long enough to ensure patients would not remember their answers on baseline. Besides, in three weeks no major changes in disease activity were expected. Patients were excluded from the retest when they experienced a change in disease activity from active to quiescent disease or vice versa according to the HBI/SCCAI.

Internal consistency was analysed by using the Cronbach’s alpha index for the Dutch IBD-F Section I and Section II. A Cronbach’s alpha value between 0.70 and 0.90 was considered to be adequate [24]. Correlation between the outcomes of total sum scores of the Dutch IBD-F at two moments in time was evaluated using the two-way random model intra-class correlation method (ICC) [24]. ICC values varies from 0 to 1 with 1 being complete correlation between two outcomes. An adequate correlation between two measurement points was expected. A value above 0.70 was considered to be acceptable. However, a value between 0.80 and 0.90 was preferred [24]. To detect outliers, internal consistency for each individual item was calculated using the two-way random model ICC as well.

## Results

### Translation process (phase 1)

The translation process was conducted from June 2021 until November 2021. Following the conduction of two independent forward translations, minor linguistic and grammatical changes were made. No changes were made in the meaning of the questions and instructions. The two forward translations were combined which resulted in a synthesized version. After translation back into English, one remarkable difference between the two backward translations and the original English scale appeared. Both backward translators translated the Dutch term for fatigue (vermoeidheid) into ‘tiredness’. In Dutch, ‘vermoeidheid’ is used for defining everyday tiredness as well as for describing fatigue as a clinical or pathological symptom. The expert committee agreed that this lack of vocabulary in the Dutch language explained the difference between the two backward translations and the original English IBD-F. Therefore, no changes were made to the Dutch concept version of the IBD-F.

### Pilot-test of the dutch IBD-F (phase 2)

Pilot-interviews were performed in November 2021. After five interviews data saturation was achieved. Participants unanimously agreed that the instruction, questions and answer options of all sections of the Dutch IBD-F scale were clear. Patients stated the scale is an excellent reflection of their experience of fatigue and the impact of fatigue on their daily lives. One patient (P2) stated: ‘*I feel like the scale will allow me to explain my fatigue more clearly to my nurse or doctor.’* Time of completion ranged from 8 to 10 min, including ‘thinking out loud’ in accordance with the TSTI method [28].

The expert committee decided to adjust three questions in Section II as a result of remarks of the participants. Question 13 was changed from ‘*My sexual relationship with my partner was affected by fatigue.*’ to ‘*My sexual life was affected by fatigue.*’ because two out of five patients stated they did not have a partner. However, these interviewees stated that fatigue affects their sexual life despite the lack of a partner. Furthermore, an adjustment to question 23 was made. ‘*Fulfilling life.*’ was translated to ‘*volwaardig leven.*’ in Dutch. However, all interviewees agreed that ‘*volwaardig leven*’ (full life) is associated with choices concerning life and death and therefore feels heavy to answer. Expert committee agreed to replace ‘*volwaardig leven*’ (full life) by ‘*bevredigend leven*’ (more close to fulfilling life). Finally, after a process audit by the developer of the original scale (WCD), no changes were made.

### Psychometric properties (phase 3)

#### Participants

**Fig. 2:**
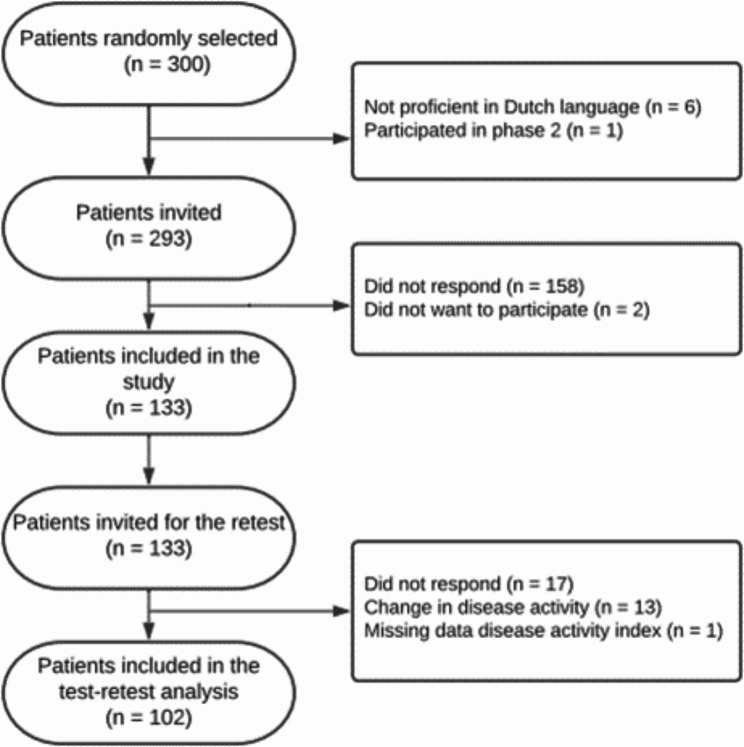
Enrolment process

The enrolment process is shown in Fig. 2. Postal invitations were sent out in December 2021. In total, 293 patients were invited of whom 133 (45%) agreed to participate and were included in the study and of whom 102 (35%) were also included in the retest analysis. Clinical characteristics of the participants at baseline and three weeks later were similar, as shown in Table 1.

**Table 1:** Clinical characteristics of participants

	Test (t = 0) (n = 133)	Retest (t = 3 weeks) (n = 102)
**Gender, n (%)**		
Male	63 (47.4)	46 (45.1)
**Age in years, median (range)**	52 (21–82)	54 (21–82)
**Condition, n (%)**		
CD	75 (56.4)	59 (57.8)
UC	53 (39.8)	39 (38.2)
IBD-U	5 (3.8)	4 (3.9)
**Disease duration in years, median (range)**	12 (1–56)	15 (1–56)
**Method of completion, n (%)**		
Electronically	55 (41.4)	44 (43.1)
**Montreal Score** ^**1**^ ** CD – age at onset, n (%)** ^**2**^		
< 16 years	6 (8.0)	6 (10.2)
17–40 years	45 (60.0)	32 (54.2)
> 40 years	24 (32.0)	21 (35.6)
**Montreal Score** ^**1**^ ** CD – disease location, n (%)** ^**2**^		
Ileal	21 (28.0)	18 (30.5)
Colonic	27 (36.0)	23 (39.0)
Ileocolonic	27 (36.0)	18 (30.5)
**Montreal Score** ^**1**^ ** CD - disease behaviour, n (%)** ^**2**^		
Nonstricturing, nonpenetrating	40 (53.3)	33 (55.9)
Stricturing	18 (24.0)	13 (22.0)
Penetrating	17 (22.7)	13 (22.0)
**Disease extension UC/IBD-U, n (%)** ^**3**^		
Proctitis	11 (19.0)	9 (20.9)
Left-sided (distal to splenic flexure)	16 (27.6)	9 (20.9)
Extensive (proximal to splenic flexure)	31 (53.4)	25 (58.1)
**Disease activity, n (%)**		
CD in remission	55 (41.4)	47 (46.1)
CD active disease	20 (15.0)	12 (11.8)
UC / IBD-U in remission	48 (36.0)	39 (38.2)
UC / IBD-U active disease	10 (7.5)	4 (3.9)

*CD = Crohn’s disease, UC = ulcerative colitis, IBD-U = inflammatory bowel disease unspecified*

^*1*^*Montreal classification of Silverberg et al. (2005)* [34]. ^*2*^*% of CD patients.*^*3*^*% of UC/IBD-U patients*

#### Convergent validity

The Dutch IBD-F scale showed a median sum score regarding the level of fatigue (Section I) of 7.5 (range 0.0–19.0). The median sum score regarding the impact of IBD fatigue (Section II) was 18.4 (range 0.0–84.0). The MFI-20 showed median scores of 12 on the general fatigue subscale, 12 on the physical fatigue subscale, 11 on the reduced activity subscale, 8 on the reduced motivation subscale and on 9 the mental fatigue subscale.

Table 2 shows the correlations for each of the sections and subscales of the IBD-F and MFI-20. All correlations were statistically significant (p < .001). The highest correlation (*r* = .86; p < .001) was between IBD-F Section I (level of fatigue) and the general fatigue subscale of the MFI-20. The Spearman’s *r* value of 0.86 indicated good correlation between the evaluation of general fatigue with the IBD-F (Section I) and the evaluation of general fatigue subscale of the MFI-20. The lowest correlation (*r* = .57; p < .001) was found between Section I (level of fatigue) of the IBD-F and the mental fatigue subscale on the MFI-20. All correlations exceed the minimum of *r* = .50 indicating good convergent validity between the IBD-F and MFI-20.

**Table 2:** Convergent validity Dutch IBD-F scale and Dutch MFI-20

	MFI-20 General fatigue	MFI-20 Physical fatigue	MFI-20 Reduced activity	MFI-20 Reduced motivation	MFI-20 Mental fatigue
**IBD-F Section I** (level of fatigue)	0.86*	0.78*	0.64*	0.59*	0.57*
**IBD-F Section II** (impact of fatigue)	0.82*	0.79*	0.63*	0.63*	0.65*

IBD-F = inflammatory bowel disease fatigue scale, MFI-20 = multidimensional fatigue inventory

* p < .001

#### Internal consistency

For Section I and Section II of the Dutch IBD-F, a Cronbach’s alpha of 0.94 and 0.97 was found respectively. Removing single individual items decreased the level of Cronbach’s alpha to 0.92 for Section I and to 0.96 for Section II. This indicated the absence of remarkable outliers affecting the internal consistency. For comparison purposes, internal consistency of the MFI-20 was calculated for each of the five subscales. The following values of Cronbach’s alpha were found: 0.87 (general fatigue), 0.84 (physical fatigue), 0.79 (reduced activity), 0.74 (reduced motivation) and 0.83 (mental fatigue).

#### Reliability

An ICC value of 0.85 (95% CI 0.79–0.90) was found for test and retest reliability of the total sum score of Section I of the IBD-F. Test and retest reliability for the total sum score of Section II showed an ICC value of 0.88 (95% CI 0.83–0.92). Test and retest reliability values for individual items of the IBD-F are shown in Table 3.

**Table 3:** Test and retest reliability Dutch IBD-F

Section – item	Original English IBD-F	Dutch IBD-F	n	ICC (95% CI)
1.1	What is your fatigue level right now?	Hoe vermoeid bent u nu?	102	0.68 (0.56–0.77)
1.2	What was your highest fatigue level in the past two weeks?	Wat is het meest vermoeid dat u in de afgelopen twee weken bent geweest?	102	0.85 (0.78–0.90)
1.3	What was your lowest fatigue level in the past two weeks?	Wat is het minst vermoeid dat u in de afgelopen twee weken bent geweest?	101	0.63 (0.50–0.74)
1.4	What was your average fatigue level in the past two weeks?	Hoe vermoeid was u gemiddeld in de afgelopen twee weken?	102	0.78 (0.68–0.84)
1.5	How much of your waking time have you felt fatigued in the past two weeks?	Hoe vaak heeft u zich vermoeid gevoeld in de afgelopen twee weken?	102	0.77 (0.67–0.84)
**Total score Section I**			**101**	**0.85 (0.79–0.90)**
2.1	I had to nap during the day because of fatigue.	Ik moest overdag dutten door vermoeidheid.	102	0.81 (0.73–0.87)
2.2	Fatigue stopped me from going out to social events.	Vermoeidheid weerhield me ervan om aan sociale gelegenheden deel te nemen.	102	0.81 (0.72–0.86)
2.3	I was not able to go to work or college because of fatigue.	Ik kon niet naar werk of school gaan door vermoeidheid.	64*	0.59 (0.41–0.73)
2.4	My performance at work or education was affected by fatigue.	Mijn prestaties op werk of school werden beïnvloed door vermoeidheid.	63*	0.61 (0.43–0.75)
2.5	I had problems concentrating because of fatigue.	Ik had concentratieproblemen door vermoeidheid.	102	0.79 (0.70–0.85)
2.6	I had difficulty motivating myself because of fatigue.	Ik had moeite om mezelf te motiveren door vermoeidheid.	102	0.75 (0.65–0.82)
2.7	I could not wash and dress myself because of fatigue.	Ik kon mezelf niet wassen of aankleden door vermoeidheid.	101	0.60 (0.46–0.71)
2.8	I had difficulty with walking because of fatigue.	Ik had moeite met lopen door vermoeidheid.	101	0.67 (0.55–0.77)
2.9	I was unable to drive as much as I need to because of fatigue.	Ik kon niet zo veel autorijden als nodig was door vermoeidheid.	82*	0.44 (0.24–0.57)
2.10	I was not able to do as much physical exercise as I wanted to because of fatigue.	Ik kon niet zo veel sporten als ik wilde door vermoeidheid.	98	0.74 (0.63–0.82)
2.11	I had difficulty continuing with my hobbies/interests because of fatigue.	Ik had moeite om mijn hobby’s/interesses uit te voeren door vermoeidheid.	102	0.71 (0.60–0.73)
2.12	My emotional relationship with my partner was affected by fatigue.	De emotionele relatie met mijn partner werd beïnvloed door vermoeidheid.	83*	0.56 (0.40–0.69)
2.13	My sexual relationship with my partner was affected by fatigue.	Mijn seksuele leven werd beïnvloed door vermoeidheid.	81*	0.65 (0.51–0.73)
2.14	My relationship with my children was affected by fatigue.	De relatie met mijn kinderen werd beïnvloed door vermoeidheid.	67*	0.73 (0.59–0.83)
2.15	I was low in mood because of fatigue.	Ik had een slechte stemming door vermoeidheid.	102	0.69 (0.57–0.78)
2.16	I felt isolated because of fatigue.	Ik voelde me geïsoleerd door vermoeidheid.	102	0.73 (0.62–0.81)
2.17	My memory was affected because of fatigue.	Mijn geheugen werd beïnvloed door vermoeidheid.	102	0.80 (0.72–0.86)
2.18	I made mistakes because of fatigue.	Ik maakte fouten door vermoeidheid.	102	0.61 (0.47–0.72)
2.19	Fatigue made me irritable.	Vermoeidheid maakte me prikkelbaar.	102	0.78 (0.70–0.85)
2.20	Fatigue made me frustrated.	Vermoeidheid maakte gefrustreerd.	102	0.77 (0.68–0.84)
2.21	I got words mixed up because of fatigue.	Ik haalde woorden door elkaar door vermoeidheid.	101	0.64 (0.51–0.74)
2.22	Fatigue stopped me from enjoying life.	Vermoeidheid weerhield me ervan om van het leven te genieten.	102	0.68 (0.56–0.78)
2.23	Fatigue stopped me from having a fulfilling life.	Vermoeidheid weerhield me van het leiden van een bevredigend leven.	102	0.75 (0.65–0.82)
2.24	My self-esteem was affected by fatigue.	Mijn zelfbeeld werd beïnvloed door vermoeidheid.	102	0.80 (0.72–0.86)
2.25	Fatigue affected my confidence.	Vermoeidheid beïnvloedde mijn zelfvertrouwen.	102	0.65 (0.52–0.75)
2.26	Fatigue made me feel unhappy.	Vermoeidheid maakte dat ik me ongelukkig voelde.	102	0.70 (0.59–0.79)
2.27	I had difficulties sleeping at night because of fatigue.	Ik had ’s nachts moeite met slapen door vermoeidheid.	101	0.67 (0.54–0.76)
2.28	Fatigue affected my ability to do all my normal household activities.	Door vermoeidheid was ik niet in staat om al mijn huishoudelijke taken uit te voeren.	102	0.69 (0.60–0.78)
2.29	I had to ask others for help because of fatigue.	Ik moest anderen om hulp vragen vanwege vermoeidheid.	101	0.62 (0.48–0.73)
2.30	Quality of my life was affected by fatigue.	Mijn kwaliteit van leven werd beïnvloed door vermoeidheid.	102	0.66 (0.53–0.75)
**Total score Section II**			**98**	**0.88 (0.83–0.92)**

IBD-F = inflammatory bowel disease fatigue self-assessment scale, ICC = intraclass correlation coefficient. * N/A answer option applies for these items

Furthermore, test and retest reliability was calculated for total sum scores of each subscale of the MFI-20. The following ICC values were found: ICC 0.82 (95% CI 0.75–0.88) for general fatigue, ICC 0.79 (95% CI 0.70–0.85) for physical fatigue, ICC 0.72 (95% CI 0.61–0.80) for reduced activity, ICC 0.71 (95 CI 0.60–0.80) for reduced motivation and ICC 0.72 (95% CI 0.61–0.80) for mental fatigue.

## Discussion

The aim of this study was to translate the English version of the IBD-F into Dutch and to validate the Dutch IBD-F scale. In line with the results of the validation study of the original English IBD-F, Dutch patients stated that the Dutch IBD-F scale adequately reflected their experience of fatigue and the impact of this fatigue on their daily lives [5]. Cronbach’s alpha values for internal consistency of the Dutch IBD-F were high and nearly equal to the Cronbach’s alpha values of the original IBD-F. These high values might reveal a tendency towards redundancy of items and therefore a reduction of items could be considered [35, 36]. However, further research is required to identify which items, if any, could be removed. In clinical practice, it is clearly preferable to apply short patient-reported outcome measures (PROMs) with adequate psychometric properties rather than longer scales, as shorter PROMs reduce the effort for patients completing the scale, which in turn may lead to increased response rates [37, 38]. Though, the time of completion of the Dutch IBD-F scale was only 8–10 min. This can be done by the patient at home before clinical consultation.

In the present study, a high correlation was found between test and retest, indicating that the test-retest reliability of the Dutch IBD-F is adequate. However, one notable outlier was observed: question 9 in Section II of the IBD-F showed poor test-retest reliability. This item is a question regarding driving. A possible reason for the poorer test-retest reliability may be the fact that the postal invitation for the baseline scales was sent right before the Christmas holidays and the retest was completed three weeks later. The necessity to drive may have differed between those measurement moments.

A great strength of the study was the fact that the design is in line with the widely accepted COSMIN guidelines [26, 27]. Furthermore, the validity and reliability of the study results were clearly increased by the fact that the pilot-test was done with the TSTI method, a well justified method for qualitative research, and by the large sample size in phase 3. Additionally, the high response rate for the retest was beneficial for the reliability of the study outcomes [26–28, 39].

There are some potential drawbacks associated with our study. The fact that patients with comorbidities were not excluded from the current study might be considered a limitation. The presence of other diseases may have contributed to the level of fatigue measured. However, the aim of this study was to validate the translated version of the IBD-F in a Dutch IBD population. In clinical practice, comorbidities are common in patients with IBD and may therefore not be ignored [40]. An additional analysis corrected for comorbidities might improve the understanding of the level of fatigue that was particularly caused by IBD itself.

Although we complied to the COSMIN guidelines by conducting 5 interviews in the pilot test and achieving data saturation, we did not comply to the 30–40 patients recommended by the guidelines of Beaton et al. [25, 26]. We aimed to achieve heterogeneity in the sample by selecting patients purposively according to demographic and disease specific characteristics. However, due to the small sample size it might not have fully reflected the Dutch IBD population.

While the present study thoroughly evaluated convergent validity, it did not assess discriminative validity. This assessment might be important to establish that the Dutch IBD-F measures fatigue and not another construct. For instance, fatigue in patients with IBD is associated with anxiety and depression [3]. To examine whether the Dutch IBD-F measures fatigue and not anxiety nor depression, future studies might assess discriminative validity, e.g. by evaluating correlation between the Dutch IBD-F and the Hospital Anxiety and Depression Scale (HADS) [41].

In the current study, the Dutch IBD-F was not translated from a Flemish perspective. To adequately apply the Dutch IBD-F in a Flemish population, further research ought to be considered. There may be minor linguistic differences between Dutch and Flemish like this was found in a previous cross-cultural validation study [42].

A notable finding is the fact that sum scores of the Dutch IBD-F were lower than the sum scores found in the original validation study [5]. This discrepancy may be due to the fact that data were collected during the COVID-19 pandemic while there was a lockdown in the Netherlands. During the process of data collection, gymnasia, schools and leisure facilities were closed. The impact of fatigue may have been underestimated because, for example, working out or going to school was not possible due to these COVID-19 measures. Particularly in Section II of the IBD-F, which evaluates the impact of fatigue on patients’ daily lives, the sum score may have been expected to differ during a lockdown and without COVID-19 measures. A retest in a period without COVID-19 measures could be considered.

Our research showed that the Dutch IBD-F is a self-assessment scale with adequate psychometric properties to measure fatigue in Dutch patients with IBD. Using this PROM in clinical practice may contribute to the shared decision-making process by assessing fatigue from the patient’s perspective. The use of PROMs in clinical practice could facilitate bridging the gap between the physicians’ global assessments and IBD patients’ perceptions of IBD fatigue [38].

Besides applying the Dutch IBD-F in clinical practice, the Dutch IBD-F may facilitate research regarding fatigue in Dutch patients with IBD. It should, however, be noted that sensitivity to change has not been evaluated for the Dutch IBD-F nor for the original IBD-F [5]. In order to use the Dutch IBD-F for evaluation purposes or research concerning treatment strategies, additional research regarding responsiveness is required to assess the PROM’s ability to measure change over time.

## Conclusion

The thorough translation process resulted in a comprehensible, valid and reliable version of the Dutch IBD-F scale. Convergent validity with the MFI-20 appeared to be good. Furthermore, this study found excellent internal consistency and good test-retest reliability. The Dutch version of IBD-F scale was shown to have very good psychometric properties and can be used in clinical practice to provide insight into patients’ perspectives on fatigue experienced. Additional research is recommended in order to use the Dutch IBD-F for evaluation purposes and to examine the possibility of reducing the number of items.

### Electronic supplementary material

Below is the link to the electronic supplementary material.

Appendix 1 Interview guide

## Data Availability

The datasets used and analysed during the current study are available from the corresponding author on reasonable request.

## List of Abbreviations

CD: Crohn’s disease
CI: Confidence interval
FACIT-F: Functional Assessment of Chronic Illness Therapy-Fatigue
FQ: Fatigue Questionnaire
HBI: Harvey Bradshaw Index
HRQoL: Health-related quality of life
IBD: Inflammatory Bowel Disease
IBD-F: Inflammatory Bowel Disease Fatigue patient self-assessment scale
IBD-U: Inflammatory Bowel Disease unspecified
ICC: Intra-class correlation
MFI-20: Multidimensional Fatigue Inventory
N-ECCO: Nurses European Crohn’s and Colitis Organisation
PROMs: Patient-reported outcome measures
SCCAI: Simple Clinical Colitis Activity Index
SD: Standard deviation
TSTI: Three step test interview
UC: Ulcerative colitis
