# Supplementary material for: Translation, validation and psychometric properties of the Dutch version of the Inflammatory Bowel Disease-Fatigue (IBD-F) self-assessment scale
Source: J Patient Rep Outcomes. 2023 Oct 30;7:108. doi: 10.1186/s41687-023-00642-3 (PMC10616031; doi:10.1186/s41687-023-00642-3)
Supplement: Supplementary file 1 — Appendix 1 Interview guide [file 41687_2023_642_MOESM1_ESM.docx]

# Appendix 1 – Interview guide

Gender:

Age:

Education level:

Disease type:

Disease duration:

Explaining the aim of the study and interview

The aim of the study is to implement a questionnaire that measures fatigue in patients with inflammatory bowel disease. The questionnaire was developed in the United Kingdom and has been translated into Dutch. With this study, we would like to examine whether the questionnaire is comprehensible in Dutch for the patients who will fill it out. So in the end, it is not so much about the outcome of the questionnaire, but rather how the completion of the questionnaire is experienced. I would like to ask you to complete all the questions in the questionnaire, if something is not applicable in your current situation, 'not applicable' may be filled in. When completing the questionnaire, I would like to ask you to think out loud. What do you think when you read the question? Is the question clear? If something is not clear, what do you have doubts about?

To try out thinking out loud, I would like to ask you to practice this with the question below.

"In general, how would you rate your satisfaction with your social activities and relationships?"

General questions:

- Are the instructions clear?

- If not, what is not clear?

- Do you have any suggestions to make it clearer?

Questions per item:

- Is the question clear?

- If not, what is not clear? What would make it clearer for you?

- Describe in your own words what you think the question means.

- Are the answer options clear?

- If not, what is not clear? What would make it clearer for you?

Closing:

- Do you have any other comments on the questionnaire?
